# The temperature of connection: psychological mechanisms and happiness generation in AI-enabled neighborhoods

**DOI:** 10.3389/fpsyg.2026.1742789

**Published:** 2026-03-11

**Authors:** Guanxi Chen, Jiajun Chen

**Affiliations:** 1 NingboTech University, Ningbo, China; 2 Joongbu University, Geumsan-gun, Republic of Korea; 3 Nantong Institute of Technology, Nantong, China

**Keywords:** AI-empowered neighborhoods, China, community well-being, continued use intention, digital attachment

## Abstract

**Introduction:**

Artificial intelligence (AI) is rapidly reshaping neighborhood services, yet we still lack a nuanced understanding of which experiential cues matter, how they combine, and why they relate to residents’ well-being. This study addresses that gap by identifying designable service cues and clarifying their socio-psychological pathways to well-being in AI-enabled neighborhoods.

**Methods:**

Drawing on the Cognition–Affect–Conation framework and insights from technology acceptance, service quality, and social presence research, we conceptualized five experiential cues: reliability, perceived usefulness, perceived ease of use, service efficiency, and aesthetic perception. We further positioned digital attachment and continued use intention as key socio-psychological linkages to well-being. Empirically, we used a three-wave survey design with four-week lags across four smart-community pilots in Zhejiang, China (matched panel *n* = 452). An explanation–prediction–necessity strategy was adopted by triangulating PLS-SEM, artificial neural networks (ANN), and necessary condition analysis (NCA).

**Results:**

Findings converged across methods. Instrumental appraisals—especially reliability, perceived usefulness, and perceived ease of use—formed the foundational conditions for digital attachment and continued engagement, which were strongly associated with higher well-being. Service efficiency and aesthetic perception acted mainly as amplifiers rather than stand-alone drivers. NCA results indicated that no single condition reached necessity, suggesting that residents’ well-being emerges from complementary combinations of experiential cues rather than any one dominant factor.

**Discussion:**

The findings suggest that well-being in AI-enabled neighborhoods is generated through configurational and pathway-based mechanisms rather than isolated service attributes. Practically, neighborhood service design should prioritize baseline thresholds in reliability, usefulness, and ease of use, while treating efficiency and aesthetics as conversion accelerators. Lightweight participation loops at high-social-presence touchpoints may further strengthen attachment, sustained engagement, and downstream well-being outcomes.

## Introduction

The rapid development of artificial intelligence (AI) is reshaping not only how urban communities deliver services but also how residents experience their everyday environments. With the diffusion of generative AI (GenAI) and multimodal interaction technologies, neighborhoods are increasingly becoming hybrid socio-digital environments in which digital interfaces, governance processes, and social cues function as “environmental signals” that shape residents’ perceived control, predictability, belonging, and ultimately psychological well-being (Usama et al., 2025; Anuyah, 2024). In this sense, AI-empowered neighborhoods are not merely technology-enabled service systems; they constitute a new form of daily-life setting where human–technology interaction becomes embedded in routine community practices and may produce downstream consequences for person–environment relations and well-being (Méndez et al., 2021). According to relevant market research and industry forecasts, the smart community/smart city market is expected to maintain a growth trend through 2030, with East Asia showing particularly strong development potential driven by advances in digital infrastructure and scenario-based applications (Hassebo and Tealab, 2023).

Unlike face-to-face interactions centered on conventional community hubs, AI-empowered neighborhoods emphasize personalized, situated, and affectively resonant forms of engagement (Kou and Gui, 2020). Social Presence Theory (SPT) provides a foundational lens for understanding these socio-technical interactions between residents and AI systems. Earlier work conceptualized social presence largely as a media attribute that approximates the cues of face-to-face contexts—such as avatars, names, tone, and affective language—to explain feelings of co-presence and being understood (Biocca et al., 2003). More recent information-systems research advances an interactional perspective: social presence is not a static property of technology but a generative accomplishment of participants who, through interaction, render the virtual other “real” (Schultze and Brooks, 2019; Guo et al., 2022).

Building on these perspectives, we conceptualize AI systems in neighborhoods as part of the ambient socio-digital environment that residents continuously interpret and respond to. Accordingly, this study examines how GenAI-enabled neighborhood experiences shape residents’ psychological connection to the digital neighborhood environment (digital attachment) and, through sustained engagement, contribute to well-being. This framing foregrounds an environmental-psychological question: how do digitally mediated neighborhood settings cultivate (or weaken) person–environment bonds and the psychological outcomes that follow?

Place Attachment Theory (PAT) offers a complementary framework for explaining the durability of these affective bonds. PAT holds that sustained interaction with particular settings fosters emotional attachment and identification (Erfani, 2022). In digitally infused environments, this attachment extends to digital place attachment, reflecting emergent modes of belonging within hybrid virtual–physical contexts (Oleksy et al., 2023). When residents continually interact and share experiences in virtual environments, digital attachment can become a pivotal determinant of subsequent behavioral intentions (Xiang et al., 2022). Extant scholarship typically conceptualizes digital attachment as a stable emotion–identity linkage between individuals and digital objects (e.g., avatars, virtual goods, digital collectibles, or social-media personae). This linkage goes beyond liking to encompass psychological ownership and self-extension—often carrying over from online experiences into offline life through identity transfer (Koles and Nagy, 2021).

While prior research has explored AI’s implications for community life from perspectives such as functional value, technology acceptance, or social interaction (Musikanski et al., 2020; Hsu et al., 2022), the psychological mechanisms through which GenAI influences community well-being via digital attachment remain under-specified. Three gaps stand out: (1) an overemphasis on AI’s functional affordances at the expense of its interpersonal and community-level affective value; (2) a lack of integrative analyses connecting digital attachment to community well-being; and (3) a paucity of empirical evidence on the socio-psychological consequences of AI-empowered neighborhood forms.

To address these gaps, we focus on AI-enabled neighborhood centers and examine how residents’ cognitive, affective, and conative processes within GenAI environments jointly shape community well-being. Specifically, we ask:

(1) How do key GenAI-enabled community experience factors influence residents’ digital attachment and community well-being?

(2) Does digital attachment mediate the relationship between AI experiences and community well-being?

(3) Can a Cognitive–Affective–Conative (CAC) framework be constructed and validated to reveal the psychological generative mechanisms of residents’ well-being in GenAI contexts?

We develop an integrated model that synthesizes TAM, service-quality cues (SERVQUAL), and Social Presence Theory (SPT), organized under a Cognitive–Affective–Conative (CAC) logic. The model posits that residents’ cognitive appraisals of AI-empowered neighborhood experiences (e.g., perceived usefulness, perceived ease of use, reliability, service efficiency, and aesthetic perception) foster affective bonding with the digital neighborhood environment (digital attachment), which in turn promotes continued use intention and contributes to well-being.

Methodologically, we adopt a multi-method strategy to strengthen inference through complementary evidence. First, we use PLS-SEM to test the hypothesized linear relationships among GenAI-enabled neighborhood experience cues, residents’ digital attachment, continued use intention, and well-being, including the associated mediation effects. Second, we employ artificial neural networks (ANN) to capture potential nonlinear patterns and to estimate predictors’ relative predictive contributions to well-being. Third, we apply necessary condition analysis (NCA) to examine whether any antecedent constitutes a necessary constraint for achieving high well-being, thereby distinguishing “influential predictors” from “indispensable conditions.”

This study contributes along three fronts. Theoretically, it integrates complementary perspectives (TAM, service-quality cues, and social presence) under a Cognitive–Affective–Conative (CAC) logic to deepen understanding of how residents’ well-being is generated in AI-empowered neighborhoods through the pathway from cognitive appraisals to affective bonding and continued engagement. Conceptually, the study extends environmental psychology into AI-enabled neighborhood settings by specifying how socio-digital environmental cues shape person–environment bonds (digital attachment) and well-being. Methodologically, the combined PLS-SEM–ANN–NCA design moves beyond the linear constraints of single-model approaches by jointly testing structural relations and mediation (PLS-SEM), capturing potential nonlinear predictive patterns and relative importance (ANN), and evaluating necessity constraints (NCA), thereby strengthening cross-method interpretive consistency. Practically, by elucidating how experience cues foster digital attachment and continued use intention that translate into well-being, the study offers an evidence-based foundation for human-centered smart-community design and resident well-being enhancement.

### Literature review and hypothesis development

This study integrates several complementary theoretical frameworks to examine how AI-empowered neighborhood experiences shape residents’ person–environment relations and well-being in everyday community life. From an environmental psychology perspective, AI-enabled neighborhood services can be conceptualized as ambient socio-digital infrastructure embedded in residents’ everyday environments, rather than isolated “technologies to adopt,” because their interface cues, interaction scripts, and governance processes operate as contextual signals that shape residents’ experience and well-being (Syamimi Masrani and Nik Husain, 2022; Dunne et al., 2021; Shin, 2025). In this sense, AI-empowered neighborhoods constitute a socio-digital setting in which residents continuously interpret environmental cues, thereby influencing perceived control, predictability, social climate, and the formation of place-related bonds. This framing resonates with emerging scholarship on sense of place in the digital age, which argues that digital layers increasingly co-constitute how people experience, evaluate, and attach to places rather than simply mediating communication (Dai and Liu, 2024). It also aligns with research on digital placemaking, where digitally mediated interactions can accelerate place attachment by shaping how residents perceive and enact their relationship with urban spaces (Canelas and Hoehnk, 2025), and with evidence that attachment dynamics condition people’s acceptance of “virtual alterations” in real settings (Oleksy et al., 2024).

Within this socio-digital environmental context, each framework illuminates a distinct stage of the Cognitive–Affective–Conative (CAC) process. The Technology Acceptance Model (TAM) explicates residents’ cognitive appraisals—namely perceived usefulness and perceived ease of use—which condition their continued engagement with AI-enabled neighborhood systems (Rafique et al., 2020). The SERVQUAL framework captures service-quality attributes salient to AI-mediated community services, reflecting residents’ holistic judgments of technical dependability and service experience (Idayati et al., 2020). From an interactional stance, Social Presence Theory (SPT) explains how interface and interaction cues (e.g., response latency, conversational feedback, and visual/narrative signals) co-construct a felt social “other” during interaction, shaping the perceived social atmosphere of the neighborhood’s socio-digital setting and facilitating affective bonding (Zhang H. et al., 2025; Zhang J. et al., 2025). Finally, Place Attachment Theory (PAT) foregrounds the durability of attachment to settings, providing a lens to conceptualize digital attachment as an emergent form of person–environment bond within digitally infused neighborhoods and its downstream implications for sustained participation and well-being (Farah et al., 2024; Shi et al., 2025).

Synthesizing these perspectives, we position TAM and SERVQUAL as cognitive antecedents capturing residents’ appraisals of socio-digital environmental quality, SPT as a bridging mechanism translating cognitive appraisals into affective connection via perceived social climate/co-presence, and PAT as the lens through which affect consolidates into stable person–environment bonds that support continued engagement and well-being. In doing so, the study advances an environmental-psychological account of how socio-digital neighborhood cues jointly structure residents’ everyday experiences and psychological outcomes in AI-empowered neighborhoods.

### Technology acceptance model (TAM)

The Technology Acceptance Model (TAM) posits perceived usefulness (PU) and perceived ease of use (PEOU) as its core determinants of technology adoption and continued use. PU captures whether a technology enhances task effectiveness and performance, whereas PEOU reflects the extent to which the technology is easy to learn and operate. Together, these cognitions form the evaluative basis of users’ judgments about intelligent systems and, via psychological pathways such as attitude, satisfaction, or trust, shape usage behaviors and loyalty intentions (Rafique et al., 2020).

In public-service contexts, TAM is widely applied to explain residents’ acceptance of intelligent community services and interactive systems. Evidence shows that when residents perceive greater usefulness—for example, more accurate information feedback, more efficient transaction processing, and more convenient public services—and higher ease of use—for example, user-friendly interfaces, natural interaction, low learning burden, and smooth operation—their satisfaction with digital services increases significantly, alongside stronger intentions for continued use and positive word-of-mouth (Kim et al., 2023).

Building on this line of work, the present study treats PU and PEOU as key cognitive antecedents. PU captures residents’ perceived benefits of AI-empowered neighborhoods in terms of efficiency and everyday convenience; PEOU reflects subjective experiences related to learning effort, comprehension costs, and operational fluency in AI interactions. Together, these cognitions constitute the foundation for subsequent affective attachment and, ultimately, community well-being.

### SERVQUAL

Originally proposed by Parasuraman et al. (1988), the SERVQUAL model comprises five dimensions—tangibles, reliability, responsiveness, assurance, and empathy—and assesses perceived gaps in service quality. It has been widely applied across hospitality, retail, and public services (Malik et al., 2020). As AI technologies diffuse into smart-community contexts—e.g., digital front desks, intelligent customer service, voice assistants, and neighborhood interaction platforms—scholars have contextually extended SERVQUAL by incorporating dimensions such as intelligence, interactivity, and aesthetics to better capture the experiential features of AI-enabled services (Cheng et al., 2025).

Aligning with the characteristics of smart communities, this study focuses on three resident-facing dimensions that most directly shape experience: reliability (REL), service efficiency (SER), and aesthetic perception (AES).

#### Reliability (REL)

Reliability denotes the stability and consistency with which systems fulfill service promises. In AI-mediated neighborhood services, it manifests as the accuracy of information pushes, timeliness of feedback, and robust operation under high concurrency or complex network conditions. High reliability strengthens residents’ trust and sense of security, reduces usage anxiety, and promotes satisfaction and well-being; conversely, delays or system failures erode trust and attachment to smart-community services (Jiang et al., 2025).

#### Service efficiency (SER)

Service efficiency captures speed, convenience, and responsiveness. In AI-empowered neighborhoods, it is reflected in low latency and high fluency for community transactions, information retrieval, and interactive communication. Empirical work suggests that perceived efficiency elevates resident satisfaction and participation intentions, thereby reinforcing psychological belonging to the community (Dwivedi et al., 2023). That said, over-automation may dilute interpersonal warmth and emotional exchange; striking an “efficiency–warmth” balance remains central to optimizing smart-community services.

#### Aesthetic perception (AES)

Aesthetics concerns the influence of perceptible visual and formal cues on service-quality evaluations, including interface visual appeal, clarity and orderliness of information layout, and the cultural localization of stylistic choices. Prior research shows that digital aesthetics can heighten immersion and enjoyment while reducing technological unfamiliarity and operational anxiety (Wang et al., 2010). In community settings, interfaces that embody local cultural symbols and neighborhood affective tones are more likely to catalyze emotional connection and identity alignment among residents.

### Cognitive–affective–conative (CAC) model

The Cognitive–Affective–Conative (CAC) model explicates the psychological progression from cognitive appraisal to affective response and, ultimately, to behavioral intention (Yang and Jiang, 2026). It posits that individuals first form cognitions—functional judgments and understandings—based on external stimuli; these cognitions then elicit affective reactions (e.g., trust, enjoyment, attachment); finally, such emotions translate into conative tendencies, such as continued participation, support, and recommendation. In this way, CAC delineates a three-stage “cognition–affect–behavior” mechanism and serves as a central framework for explaining attitude formation and behavioral intention (Li et al., 2023).

In service-experience research, CAC has been widely applied to examine how resident satisfaction, belonging, and well-being are formed. Prior studies show that cognitive service perceptions and functional evaluations significantly shape affective attachment, while positive affect further promotes community participation and enhances well-being (Gautam and Bhalla, 2024; Cao et al., 2022). This logic chain indicates that technological cognitions and social emotions are not separable silos in behavior formation but are transformed through a continuous psychological process.

Building on this foundation, we introduce the CAC model into AI-empowered neighborhood and digital-community contexts to investigate residents’ cognitive–emotional pathways to well-being in socio-digital environments. Specifically, the cognition stage comprises multidimensional perceptions of AI community services—reliability (REL), perceived usefulness (PU), perceived ease of use (PEOU), service efficiency (SER), and aesthetic perception (AES)—which jointly constitute residents’ overall judgments of AI-enabled neighborhood systems (Belanche et al., 2021; Chiang et al., 2021). The affect stage manifests as residents’ emotional responses to the digital community—such as trust, sense of belonging, and digital attachment—reflecting the psychological integration of functional and emotional appraisals of intelligent environments (Lee and Jeong, 2021). Finally, the conation stage is reflected in continued use intention and community well-being, representing the positive behavioral and psychological outcomes that accumulate from prior cognition and affect (Chen and Zhang, 2021; Hsu and Lin, 2023).

### Social presence theory (SPT)

Social Presence Theory (SPT) originated in social psychology to explain why people experience different levels of interaction and immediacy across mediated communication settings (Kreijns et al., 2022). With advances in generative AI and affective computing, SPT has been widely used to examine the emotional dimensions of human–AI interaction. Earlier research largely viewed social presence through a media-capability lens; more recent work emphasizes that presence is interactionally co-constructed by interlocutors through linguistic and behavioral cues in context, rather than being a fixed technological attribute (Don et al., 2022).

Extant studies suggest that felt presence is shaped by three interlocking factors: (1) technological readiness—including interface and interaction quality, immersion, and sensory stimulation; (2) content cues—such as appearance, motion patterns, and situational constraints that together generate realism and contextual congruence; and (3) user factors—including prior experience, mood, perceptual abilities, and self-efficacy. The synergy among these factors determines whether individuals feel that “the other is present and I am seen” (Zhang et al., 2022).

Accordingly, SPT offers a mechanistic lens for understanding how functional AI interactions in communities are transformed into subjective experiences of being understood, attended to, and connected. It thereby grounds subsequent theorization about how digital attachment may arise from such experiences and, in turn, shape continued use intentions and community well-being.

### Resident digital attachment

Digital attachment is defined as the affective bond that individuals form with “digital places” within digitized or hybrid (online–offline) community environments—such as community apps, smart terminals, and online neighborhood platforms (Alkhalifah et al., 2025). This bond accumulates through multiple layers of meaning, including long-term usage experience, length of residence, and the inscription of community cultural symbols into digital interfaces—paralleling antecedents of offline place attachment.

Departing from earlier views that treated attachment as a relatively static trait (Kim, 2021), recent research underscores its dynamic nature: attachment may strengthen or weaken as technological forms, life contexts, and platform functionalities evolve; moreover, individuals may simultaneously sustain attachments to multiple digital and offline places (Oleksy et al., 2023). Stable and positive place attachment is associated with higher well-being, reduced stress, and stronger senses of belonging and safety (Nisa et al., 2020). Over time, such bonds deepen into identity-laden forms of attachment (Trąbka, 2019). In digital settings, functional fluency and situational fit—for example, seamlessly completing routine tasks, effectively voicing opinions, and receiving timely responses—can elevate users’ self-efficacy and self-esteem (Chen and Gao, 2023; Oh et al., 2023), thereby motivating continued contact and prolonged dwelling in digital places.

Taken together, we infer that stronger digital attachment to existing AI-enabled community platforms increases residents’ willingness to keep living and interacting within the same socio-digital ecosystem and, through sustained emotional bonding and efficacy experiences, promotes community well-being. Put differently, when “digital dwelling” is subjectively perceived as superior to—or better aligned with—offline alternatives, the prospects of continuance and positive evaluations become more attractive and secure.

### Continued use intention

Continued use intention is an independent construct that reflects users’ willingness and resolve to reuse an information system. Scholars generally regard it as a post-adoption behavioral indicator and a key proxy for a system’s long-term value (Tam et al., 2020). Compared with initial acceptance, continued use intention more accurately predicts users’ enduring dependence on—and loyalty to—the platform (Jia et al., 2020).

Prior studies show that continued use intention is not only an endorsement of system functionality but also an expression of affective bonds formed through sustained interaction (Cao et al., 2022). Its formation can be viewed as a dynamic feedback loop across cognitive, affective, and social-interaction layers (Xiang et al., 2022), strengthened by users’ dual evaluations of a platform’s functional and emotional affordances (Ruan et al., 2025).

In smart-community settings, continued use intention—more than initial adoption—captures users’ long-term reliance on the platform and their adaptation of it into daily routines. Once continued use intention is established, platforms can sustain long-term engagement by enhancing participation, building responsive feedback mechanisms, and optimizing personalized experiences (Maslowska et al., 2022). Accordingly, this study treats continued use intention as both a downstream extension of digital attachment and a key driver of community well-being in AI-empowered neighborhoods.

### Variable extraction and conceptual model development

The primary aim of this study is to identify the salient variables embedded in AI-empowered neighborhood experiences and to develop a corresponding conceptual model that explains how these variables jointly shape residents’ digital attachment and community well-being. To this end, we adopt an inductive research approach that synthesizes extant literature and preliminary empirical evidence to extract AI experience–related variables and assemble them into an integrated theoretical framework.

Induction is well suited for phenomena in emerging domains that are not yet fully theorized. By proceeding from observed data, researchers can systematically surface relevant constructs and their interrelations, then build models and evaluate their theoretical applicability (Hayes et al., 2010). In smart-community research, AI experience typically spans (a) cognitive perceptions of the system (e.g., perceived usefulness, reliability, ease of use), (b) affective responses (e.g., digital attachment, trust, sense of belonging), and (c) behavioral outcomes (e.g., continued use intention, recommendation intention). These variables are interconnected rather than isolated, collectively driving residents’ psychological and behavioral responses—especially the formation of well-being in intelligent environments.

Guided by a literature review and initial empirical analyses, we therefore select variables closely associated with digital attachment, continued use intention, and community well-being: perceived usefulness (PU), perceived ease of use (PEOU), reliability (REL), service efficiency (SER), and aesthetic perception (AES). This approach clarifies construct definitions and the causal architecture among variables while providing theoretically grounded and practically actionable guidance for smart-community management.

### Hypothesis development

AI-Empowered Neighborhood Experience and Residents’ Digital Attachment.

In AI-empowered communities, residents’ cognitive appraisals of the system are foundational to the emergence of digital attachment. Prior research shows that perceived quality, functional utility, and interaction experience with intelligent services significantly shape affective responses and behavioral intentions (Fan et al., 2020). We posit that when residents perceive AI systems as reliable, useful, and easy to interact with, they are more likely to experience trust and enjoyment, thereby strengthening emotional bonding and psychological attachment to the digital community.

Accordingly, we propose:

*H1a*: Reliability has a significant positive effect on residents’ digital attachment.

*H1b*: Perceived usefulness has a significant positive effect on residents’ digital attachment.

*H1c*: Perceived ease of use has a significant positive effect on residents’ digital attachment.

*H1d*: Service efficiency has a significant positive effect on residents’ digital attachment.

*H1e*: Aesthetic perception has a significant positive effect on residents’ digital attachment.

### Residents’ digital attachment, continued use intention, and well-being

Digital attachment refers to the emotional bond residents develop with AI-empowered community platforms; it plays a pivotal role in driving continued use intention. In highly interactive, controllable digital environments, attachment built through sustained investment and psychological ownership can permeate online–offline boundaries and shape personal identity and everyday life (Koles and Nagy, 2021). Prior work highlights the motivational force of digital attachment for behavioral intentions, particularly in technology acceptance and long-term usage contexts. Emotional attachment has been shown to significantly strengthen users’ intentions to continue using technologies (Nafees and Sujood, 2025). Moreover, when users perceive that a platform satisfies their affective needs and delivers stable, fluent experiences, their continued use intentions rise markedly (Wang and Wang, 2023). Accordingly, we argue that digital attachment functions as a critical bridge between AI-enabled neighborhood cognitions and behavioral intentions:

*H2*: Residents’ digital attachment has a significant positive effect on continued use intention.

Beyond behavior, affective attachment increases trust and satisfaction while enhancing psychological safety and belonging (Pang and Zhang, 2024). In AI-empowered neighborhoods, residents who feel attached to the platform may construe it not merely as a service tool but as a “partner” intertwined with daily routines—an intimacy that can meaningfully elevate community well-being. We therefore propose:

*H3*: Residents’ digital attachment has a significant positive effect on well-being.

### Impact of continued use intention on well-being

Continued use intention reflects the extent to which residents are willing to keep interacting with AI-empowered neighborhood platforms over time. Prior research shows that when such intentions are established, users experience greater satisfaction and well-being (Huang, 2021). In AI-empowered communities, we contend that residents’ continuance is shaped not only by functional affordances but also by affective factors (e.g., digital attachment). Accordingly, we propose:

*H4*: Continued use intention has a significant positive effect on well-being.

### Chain mediation of residents’ digital attachment and continued use intention

In AI-empowered neighborhoods, residents’ continued use intention can be shaped by multiple cognitive dimensions, especially via the mediating role of digital attachment as an affective bond. First, reliability strengthens trust in the platform, which, in turn, fosters affective dependence (Piyasunthornsakul et al., 2022; Jo, 2022). Perceived usefulness increases the platform’s utilitarian value, thereby reinforcing residents’ emotional reliance on it (Li et al., 2021). Perceived ease of use lowers cognitive load during use, enhancing operational comfort and self-confidence (He, 2022). Service efficiency improves response speed and task fluency (Cheng and Jiang, 2020), which not only elevates satisfaction but also deepens emotional bonding with the platform. Finally, aesthetic perception—through visually appealing, culturally resonant interfaces—intensifies residents’ emotional investment (Zhang H. et al., 2025).

Synthesizing these arguments, we posit that digital attachment mediates the links between multiple cognitive appraisals (reliability, perceived usefulness, perceived ease of use, service efficiency, and aesthetic perception) and continued use intention; through this affect-driven pathway, residents’ intentions are strengthened and community well-being is subsequently enhanced.

*H5a*: Reliability positively influences well-being via a chain mediation of residents’ digital attachment and continued use intention.

*H5b*: Perceived usefulness positively influences well-being via a chain mediation of residents’ digital attachment and continued use intention.

*H5c*: Perceived ease of use positively influences well-being via a chain mediation of residents’ digital attachment and continued use intention.

*H5d*: Service efficiency positively influences well-being via a chain mediation of residents’ digital attachment and continued use intention.

*H5e*: Aesthetic perception positively influences well-being via a chain mediation of residents’ digital attachment and continued use intention.

**Figure 1:**
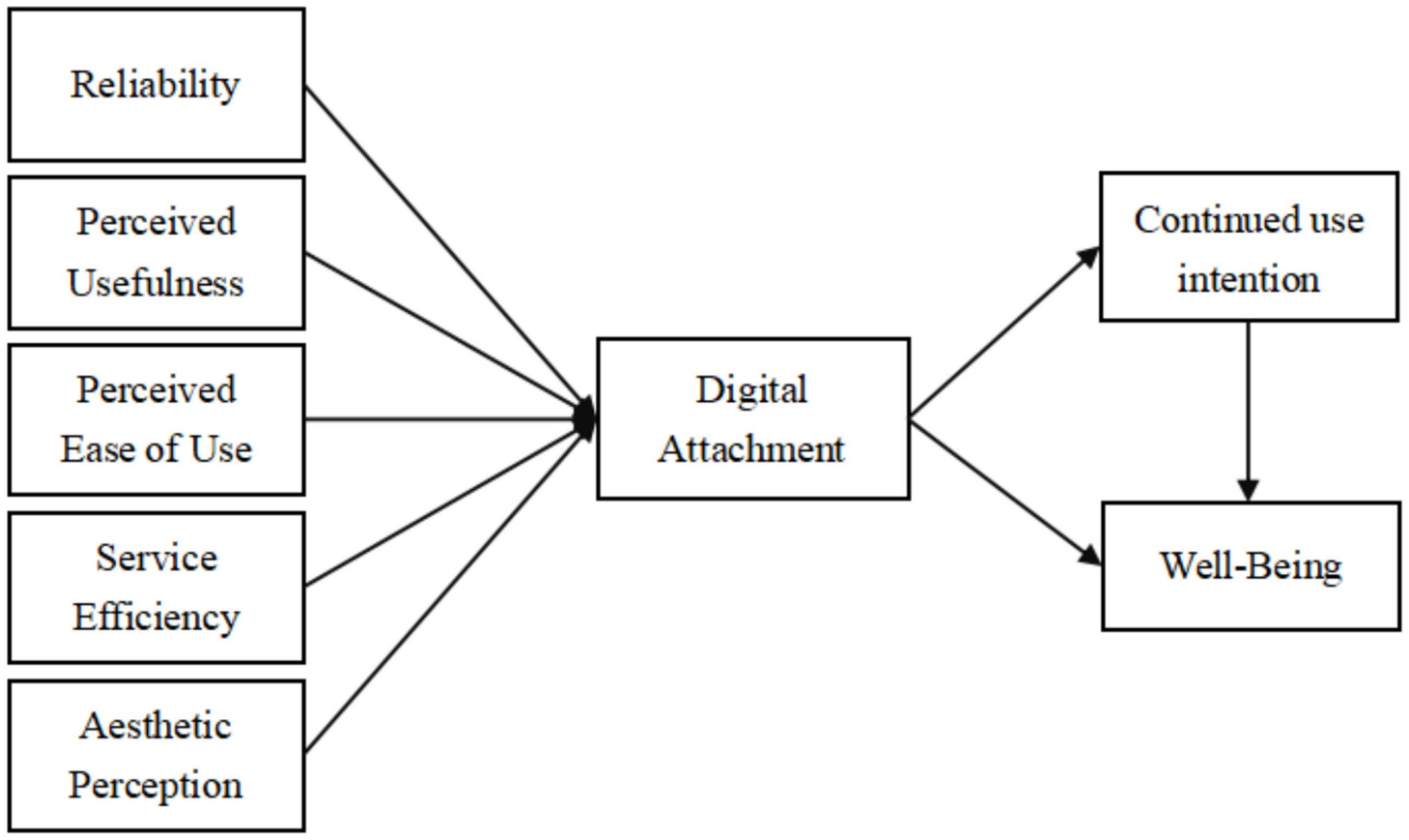
Model.

In summary, this study proposes a comprehensive conceptual model (Figure 1).

## Methods

### Case

This study examines four pilot sites in Zhejiang Province, China: Hongmei Community (Shaoxing), Meihu Future Community (Shaoxing), Haichuang Community (Ningbo), and Mingshan Community (Cixi). Each site is a representative exemplar of integrating “AI+” into community services. Centered on resident experience while emphasizing digital inclusion and multi-terminal access, these communities have built integrated platforms that connect people–places–housing–objects–organizations, with multi-scenario applications across convenience services, public security, elder care, property management, and volunteer services.

These field settings align closely with the research model. Their high-frequency touchpoints and continuous operations facilitate measurement of reliability, service efficiency, perceived usefulness, perceived ease of use, and aesthetic perception. Stable platform operation and locally tailored interfaces enable the identification of residents’ digital attachment and the observation of its downstream effects on continued use intention and well-being, including potential chain mediation. Consequently, the four pilot communities provide authentic and replicable contexts for testing the psychological mechanisms of AI-empowered neighborhood experiences and the formation of behavioral intentions.

### Research method

This study employed a three-wave longitudinal survey design targeting permanent residents of four smart-community pilots. To mitigate common method bias and align with the theorized antecedent–mediator–outcome chain, each wave was spaced 4 weeks apart. Participants were recruited via community outreach; quotas were set by the research team based on community size and demographic structure, followed by random selection. Inclusion criteria were: age ≥ 18, at least one use of a smart-community terminal or platform in the past 30 days, and signed written informed consent. Minors and individuals with evident reading/writing impairments were excluded. All measures were administered via paper questionnaires, with on-site research assistants providing instructions and answering queries.

All procedures involving human participants were conducted in accordance with relevant guidelines and regulations and complied with the Declaration of Helsinki. We confirm that this study received ethical approval from the Institute of Art and Technology Design, School of Design, NingboTech University, with approval number [NBT-ATD-EC--20250520]. Written informed consent was obtained from all participants prior to data collection. Data were collected anonymously, used solely for academic purposes, and stored separately from any potentially identifying information.

At Time 1 (T1), we measured cognitive antecedents of AI-empowered community experience—reliability (REL), perceived usefulness (PU), perceived ease of use (PEOU), service efficiency (SER), and aesthetic perception (AES)—and collected demographic and usage-behavior controls. At Time 2 (T2) (4 weeks after T1), we assessed residents’ digital attachment (RDA). At Time 3 (T3) (4 weeks after T2), we measured continued use intention (CUI) and well-being (WELL).

To anonymously match responses across waves, at T1 each participant received a paper card bearing a unique random code to be retained through T3. After each session, respondents sealed their completed questionnaire in an envelope and deposited it in a collection box. Small gifts of equivalent value were provided at each wave to compensate time costs.

#### Scale design

All instruments were contextually adapted to the smart-community setting following a pilot study and measured on a 7-point Likert scale (1 = strongly disagree, 7 = strongly agree) (Table 1).

**Table 1:** Measurement scale.

Initial dimension	Code and item description
Reliability	R1, I believe the AI neighborhood system provides accurate and reliable information.
	R2, The smart community platform rarely experiences failures or errors during operation.
	R3, When I need help, the AI system responds promptly and correctly.
Perceived usefulness	PU1, AI services effectively improve the convenience of my community life.
	PU2, Using the AI neighborhood system makes my life feel smarter and more efficient.
	PU3, I think AI services increase my life efficiency.
Perceived ease of use	PE1, I find the AI neighborhood system easy to learn and operate.
	PE2, Using AI services does not require much effort or time.
	PE3, I can easily find various functions within the AI system.
Service efficiency	SE1, The AI neighborhood system responds to my requests quickly.
	SE2, The smart community platform efficiently completes various service tasks.
	SE3, The AI system saves me time when handling community matters.
Aesthetic perception	AP1, I find the AI neighborhood system visually appealing.
	AP2, The interface design of the smart community platform gives me visual pleasure.
	AP3, The system’s colors and graphic design make me feel warm and emotionally connected.
Digital attachment	DA1I feel a strong emotional attachment to the community where I live.
	DA2, I have built important interpersonal relationships and emotional bonds here.
	DA3, I am proud to be a member of this community.
Well-being	WB1, I am satisfied with my current community life.
	WB2, Living in this AI-empowered community makes me happier.
	WB3, I often feel positive and joyful in my community life.
Continued use intention	CI1, I intend to continue using the community’s AI services/platform in the future.
	CI2, I will use this platform as my primary channel for daily community matters and interactions.
	CI3, I am willing to invest more time using the platform’s functions in the future.

Reliability (REL). Items adapted from Sumi and Kabir (2021) and Noor et al. (2022). Service Efficiency (SER). Items adapted from Altuntas et al. (2022) and Hoque et al. (2023). Aesthetic Perception (AES). Items adapted from Chang et al. (2024) and Karaca and Sarigül (2021). Perceived Usefulness (PU) and Perceived Ease of Use (PEOU). Items adapted from Akther and Nur (2022) and Park and Kim (2023). Digital Attachment (DA). Items adapted from Huang and Huang (2025) and Nafees and Sujood (2025). Continued Use Intention (CUI). Items adapted from Orden-Mejia et al. (2025). Well-Being (WB). Items adapted from Rochira et al. (2023) and Chou et al. (2022).

#### Data collection

This study employed a three-wave questionnaire design at equal intervals (every 4 weeks). Across the three waves, 1,499 questionnaires were distributed and 1,395 valid responses were returned. After three-wave matching and quality control, a final panel of 412 participants with complete data was obtained.

The sample showed a roughly balanced gender distribution; most respondents were company employees or self-employed. A majority held a bachelor’s degree or higher. The largest age group was 36–45 years, and the most common monthly income range was RMB 4,000–6,000. Detailed demographics are reported in Table 2.

**Table 2:** Frequency analysis results.

Category	Option	Frequency	%
Gender	Male	230	50.88%
	Female	222	49.12%
Occupation	Student	21	4.65%
	Government Employee	77	17.04%
	corporate Employee	195	43.14%
	self-employed	159	35.18%
Education level	High School or Below	115	25.44%
	Bachelor’s Degree	251	55.53%
	Master’s Degree or Higher	86	19.03%
Age	18–25	10	2.21%
	26–35	57	12.61%
	36–45	258	57.08%
	46–55	98	21.68%
	≥55	29	6.42%
Income	≤4,000¥	149	32.96%
	4,000–6,000¥	167	36.95%
	7,000–9,000¥	112	24.78%
	≥9,000¥	24	5.31%
Total		452	100.00%

All procedures adhered to ethical guidelines. Participants were informed of the study purpose, anonymity, and usage boundaries, and provided written informed consent. Data were used solely for academic research, stored in encrypted form, and de-identified.

### SEM–ANN–NCA method

We adopted a three-stage complementary strategy to enhance inferential robustness and explanatory power.

Stage 1: PLS-SEM (linear sufficiency). We estimated the measurement and structural models using PLS-SEM under medium-sample and potentially non-normal conditions to test linear causal paths. Reliability (REL), perceived usefulness (PU), perceived ease of use (PEOU), service efficiency (SER), and aesthetic perception (AES) served as antecedents; continued use intention (CUI) and well-being (WB) were outcomes. We report reliability and validity diagnostics, multicollinearity checks, path significances, global model fit, and robustness tests.

Stage 2: ANN (nonlinearity and non-compensatory effects). To capture nonlinear relationships and potential non-compensatory patterns, we fed the latent variable scores from PLS-SEM into a feedforward neural network to predict CUI and WB. Overfitting was controlled via cross-validation and early stopping. We computed sensitivity/permutation-based importance scores to quantify the relative contributions of REL, PU, PEOU, SER, AES, and digital attachment (DA), thereby externally calibrating the linear rank order obtained from PLS-SEM.

Stage 3: NCA (necessity thresholds). To answer the “necessity” question, we employed Necessary Condition Analysis (NCA), estimating ceiling lines and necessity effect sizes to produce bottleneck tables that indicate the minimum antecedent levels required to achieve target levels of CUI and WB.

Taken together, the three methods provide convergent and complementary evidence along linear sufficiency → nonlinear prediction → necessity thresholds, jointly elucidating the mechanism AI experience (REL/PU/PEOU/SER/AES) → DA → CUI → WB.

## Results

### Data analysis and hypothesis testing

Hypothesis testing was conducted using partial least squares structural equation modeling (PLS-SEM) in SmartPLS 3.0 (Hair et al., 2020).

### Measurement model assessment

#### Reliability and convergent validity

Cronbach’s *α*, factor loadings, AVE, and CR all met the recommended thresholds, indicating reliable internal consistency and convergent validity for this study (Table 3).

**Table 3:** Cronbach’s α, AVE and CR values.

Latent variable	Observed variable	Factor loading	Cronbach’s alpha	CR	AVE
AP	AP1	0.863	0.824	0.895	0.739
	AP2	0.837			
	AP3	0.879			
CI	CI1	0.879	0.817	0.891	0.732
	CI2	0.833			
	CI3	0.854			
DA	DA1	0.871	0.82	0.893	0.735
	DA2	0.851			
	DA3	0.851			
PE	PE1	0.843	0.816	0.89	0.729
	PE2	0.86			
	PE3	0.859			
PU	PU1	0.855	0.791	0.878	0.705
	PU2	0.844			
	PU3	0.82			
R	R1	0.864	0.818	0.891	0.732
	R2	0.831			
	R3	0.871			
SE	SE1	0.876	0.81	0.887	0.725
	SE2	0.847			
	SE3	0.83			
WB	WB1	0.866	0.818	0.892	0.733
	WB2	0.853			
	WB3	0.85			

#### Discriminant validity

The results show that the HTMT values for all constructs are below 0.85, indicating good discriminant validity among the variables (Table 4).

**Table 4:** Discriminant validity.

Construct	AP	CI	DA	PE	PU	R	SE	WB
AP								
CI	0.421							
DA	0.543	0.487						
PE	0.341	0.47	0.548					
PU	0.467	0.434	0.546	0.428				
R	0.478	0.404	0.547	0.398	0.501			
SE	0.359	0.438	0.48	0.369	0.414	0.44		
WB	0.343	0.446	0.494	0.408	0.405	0.331	0.315	

### Common method bias

We applied a full collinearity assessment to test for common method bias. The VIF values of all observed variables ranged from 1.604 to 2.037, each below 3, indicating no multicollinearity among the measures—and thus no serious common method bias.

### Structural model evaluation

**Figure 2:**
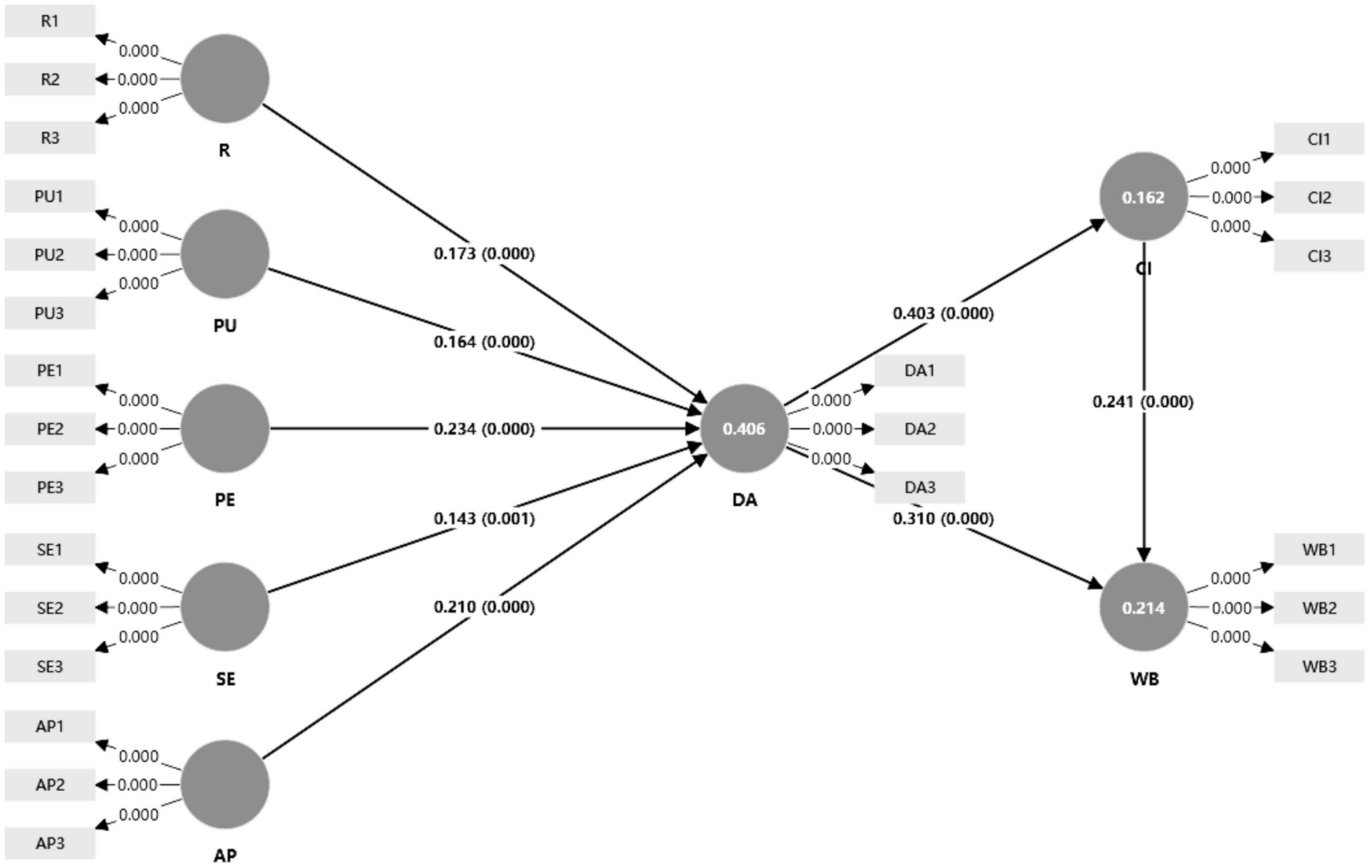
Structural equation modeling analysis results.

This study employed SmartPLS 5 to conduct structural equation modeling (SEM); the specific model specification and results are presented in Figure 2.

#### Model fit evaluation

Model fit was assessed using *R*^2^, *Q*^2^, and SRMR. As shown in Table 5, the model explains a substantial proportion of variance in digital attachment (DA; *R*^2^ = 0.406), whereas the explained variance is modest for continuance intention (CI; *R*^2^ = 0.162) and well-being (WB; *R*^2^ = 0.214). This pattern suggests that CI and especially WB are likely shaped by additional psychological, contextual, and life-course determinants beyond the current model scope; therefore, the model should be interpreted as a mechanism-focused and design-oriented explanatory slice rather than a comprehensive account of well-being. All *Q*^2^ values are greater than 0 (CI = 0.198; DA = 0.386; WB = 0.155), indicating acceptable predictive relevance for the endogenous constructs. The SRMR is 0.050, below the conventional threshold of 0.08, suggesting an adequate overall model fit (Table 5).

**Table 5:** Model fit evaluation.

Endogenous construct	*R* ^2^	*Q* ^2^
CI	0.162	0.198
DA	0.406	0.386
WB	0.214	0.155

#### Path analysis

This study used SmartPLS to analyze path effects and test the proposed hypotheses. The results are as follows (Table 6).

**Table 6:** Path analysis.

Path	*β*	SE	*t*	*p*	5.00%	95.00%
AP → DA	0.21	0.049	4.304	0	0.129	0.288
CI → WB	0.241	0.047	5.099	0	0.167	0.32
DA → CI	0.403	0.041	9.95	0	0.336	0.468
DA → WB	0.31	0.048	6.463	0	0.229	0.387
PE → DA	0.234	0.044	5.296	0	0.163	0.309
PU → DA	0.164	0.047	3.488	0	0.088	0.241
R → DA	0.173	0.043	3.999	0	0.099	0.241
SE → DA	0.143	0.044	3.267	0.001	0.071	0.217

The PLS-SEM results show that AP significantly predicts DA (*β* = 0.21, *p* < 0.05), supporting H1e. DA significantly predicts CI (*β* = 0.403, *p* < 0.05) and WB (*β* = 0.31, *p* < 0.05), supporting H2 and H3. CI also significantly predicts WB (*β* = 0.241, *p* < 0.05), supporting H4. In addition, PE (*β* = 0.234, *p* < 0.05), PU (*β* = 0.164, *p* < 0.05), R (*β* = 0.173, *p* < 0.05), and SE (*β* = 0.143, *p* < 0.05) all significantly predict DA, supporting H1a–H1d.

These results support the hypothesized directional relationships, while statistical significance should be interpreted alongside effect magnitudes and the broader methodological scope.

#### Mediation effects

This study used SmartPLS to test the mediation effects. The results are as follows (Table 7).

**Table 7:** Mediation effect analysis.

Path	*β*	SE	*t*	*p*	5.00%	95.00%
AP → DA → CI → WB	0.02	0.007	2.961	0.002	0.011	0.033
PE → DA → CI → WB	0.023	0.007	3.272	0.001	0.013	0.035
PU → DA → CI → WB	0.016	0.006	2.672	0.004	0.007	0.027
SE → DA → CI → WB	0.014	0.006	2.471	0.007	0.006	0.024
R → DA → CI → WB	0.017	0.006	2.791	0.003	0.008	0.028

All five chain mediations were significant: AP → DA → CI → WB = 0.020 (95% CI [0.011, 0.033]), PE → DA → CI → WB = 0.023 ([0.013, 0.035]), PU → DA → CI → WB = 0.016 ([0.007, 0.027]), SE → DA → CI → WB = 0.014 ([0.006, 0.024]), and R → DA → CI → WB = 0.017 ([0.008, 0.028]); consequently, H5e, H5c, H5b, H5d, and H5a are all supported.

These significant indirect effects provide evidence consistent with the proposed sequential mechanism, without implying that the outcome is determined by any single pathway alone.

### ANN results

In the second stage of the empirical analysis, we incorporated an artificial neural network (ANN), i.e., an information-processing model inspired by biological neural systems. We employed a feedforward multi-layer perceptron (MLP) architecture (input layer–one or more hidden layers–output layer) trained and evaluated via backpropagation (BP). Compared with traditional regression, ANNs do not assume normality or linear additivity and can represent nonlinear, non-compensatory mappings, thereby offering a strong complement to PLS-SEM.

**Figure 3:**
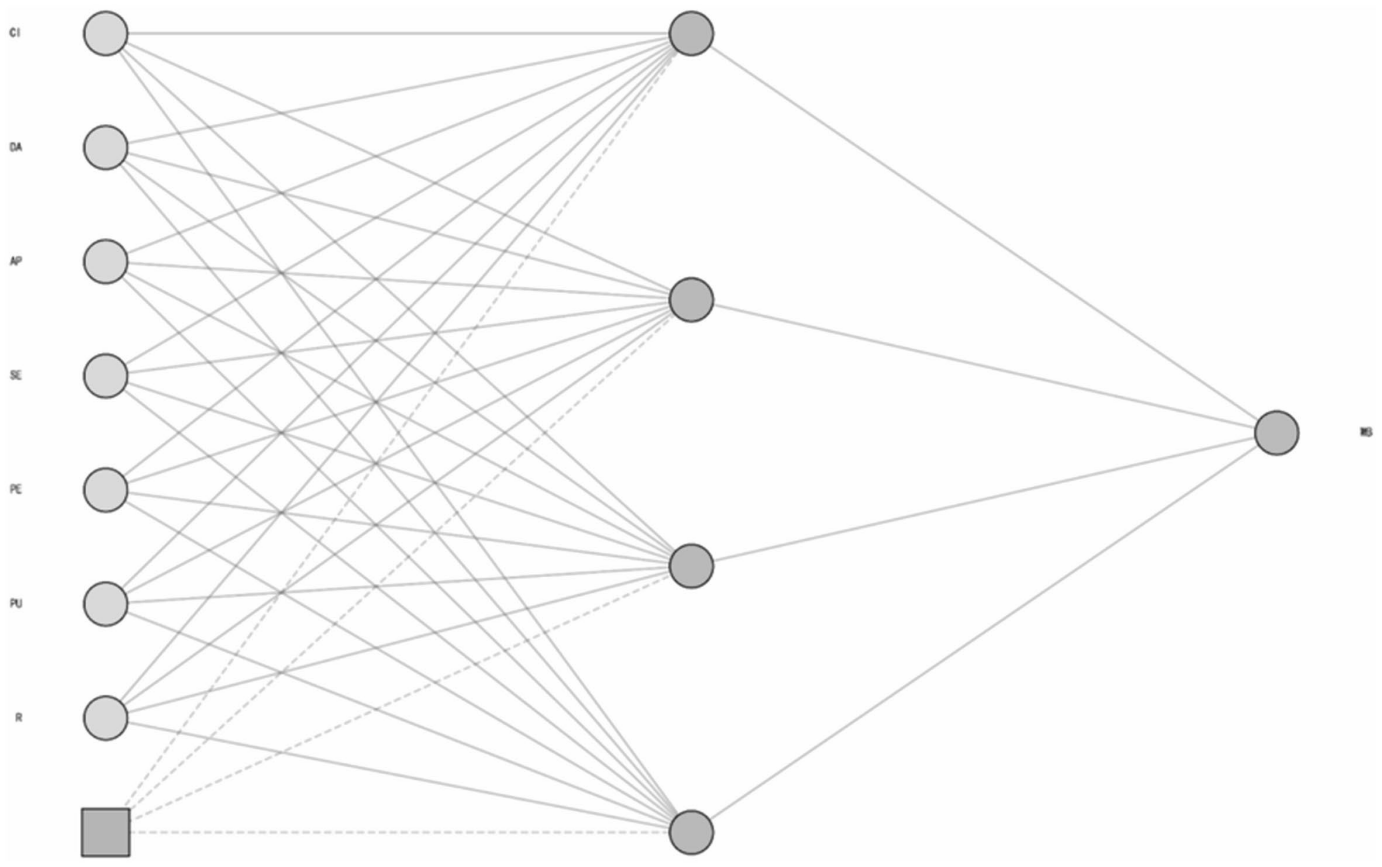
ANN model.

As shown in Figure 3, we implemented a BP-based multi-layer perceptron (MLP): the input layer comprised R, PU, PE, SE, AP, DA, and CI, the output layer was WB, and hidden layers used sigmoid activation. All variables were standardized; training used 10-fold cross-validation (~90% train, 10% test) with early stopping to curb overfitting. After training, permutation-based sensitivity analysis was used to estimate each input’s relative predictive contribution to WB, providing a nonlinear predictive complement to the PLS-SEM results.

As shown in Table 8, the mean RMSE for both training and testing is 0.105, with very small variability across folds (SD reported in Table 8; minimal dispersion indicates stable performance and consistent generalization).

**Table 8:** Predictive accuracy of the ANN model.

Neural network	Training		Testing	
	*N*	RMSE	*N*	RMSE
ANN1	298	0.107	120	0.102
ANN2	295	0.105	123	0.108
ANN3	301	0.101	117	0.109
ANN4	296	0.104	122	0.101
ANN5	300	0.110	118	0.103
ANN6	296	0.100	122	0.108
ANN7	299	0.102	119	0.101
ANN8	294	0.107	124	0.106
ANN9	298	0.105	120	0.107
ANN10	303	0.110	115	0.104
Mena		0.105		0.105
SD		0.00		0.00

As shown in Table 9, the ANN sensitivity analysis reports each antecedent’s predictive contribution to well-being (WB). Ranked by mean relative importance: digital attachment (DA) is highest, followed by continued use intention (CI); next are perceived ease of use (PE) and perceived usefulness (PU); aesthetic perception (AP) is mid-range; reliability (R) is slightly lower; and service efficiency (SE) is the weakest. Importantly, these importance scores reflect relative predictive contribution within the ANN rather than causal effect size. Nevertheless, the ordering is broadly consistent with the PLS-SEM pattern, highlighting the salience of the “attachment → continuance” pathway for WB, while purely procedural efficiency contributes comparatively less, thereby strengthening interpretive consistency across methods (Table 10).

**Table 9:** Sensitivity analysis.

Neural network	R	PU	PE	SE	AP	DA	CI
ANN1	0.2687	0.3214	0.3249	0.2514	0.279	0.4013	0.3694
ANN2	0.2673	0.3242	0.3217	0.2506	0.2763	0.4045	0.3677
ANN3	0.2698	0.3213	0.3304	0.2546	0.2769	0.4055	0.3678
ANN4	0.2688	0.3271	0.3294	0.2536	0.2769	0.4012	0.3623
ANN5	0.2659	0.3264	0.3289	0.2501	0.2794	0.4027	0.3666
ANN6	0.2663	0.3265	0.3285	0.2547	0.2792	0.4057	0.3656
ANN7	0.2707	0.3245	0.3248	0.2515	0.2837	0.4016	0.3661
ANN8	0.2715	0.3239	0.3305	0.2515	0.2826	0.4076	0.3643
ANN9	0.2729	0.323	0.3223	0.2516	0.2816	0.4039	0.3698
ANN10	0.2697	0.324	0.322	0.2536	0.2814	0.406	0.3699
Mean relative importance	0.26916	0.32423	0.32634	0.25232	0.2797	0.404	0.36695
Normalized relative importance	84.60%	82.10%	86.00%	79.10%	80.80%	69.00%	66.10%

**Table 10:** Comparison between PLS-SEM and ANN results.

Predictor	ANN importance (%)	SEM path coefficient	Importance rank
DA	0.62	0.496	6
CI	0.83	0.581	4
PE	0.83	0.747	3
PU	0.86	0.602	2
AP	0.65	0.325	5
R	0.53	0.449	7
SE	0.89	0.311	1

### NCA results

Following NCA decision rules—significance under CE-FDH or CR-FDH with *p* < 0.05 and *d* > 0 (Dul et al., 2020)—the Table 11 shows that R, PU, PE, SE, DA, and CI are often significant under both CE and CR (e.g., SE *d* ≈ 0.015, *p* = 0.003; DA *d* ≈ 0.045, *p* = 0.020; PU/PE *d* ≈ 0.011–0.013, *p* ≤ 0.044; R *d* ≈ 0.003, *p* = 0.010; CI *d* ≈ 0.014, *p* = 0.012), while AP is not significant (*p* = 0.103). Importantly, the necessity effect sizes are small overall (approximately *d* ≈ 0.001–0.045), and the bottleneck levels are low/close to zero, suggesting that—even when statistically significant—these conditions impose only weak necessity constraints rather than “must-have” thresholds. Therefore, while PLS-SEM and ANN highlight DA and CI as influential predictors of WB, the NCA results indicate that they should not be interpreted as indispensable conditions for achieving high WB. Taken together, the evidence is more consistent with a multi-factor, complementary combination logic than with a single-condition necessity account.

**Table 11:** NCA results.

Condition	Method	Accuracy (%)	Ceiling zone	Range	Effect Size (d)	*p*-value
R	CE	100.00%	0.003	0.890	0.003	0.010
	CR	100.00%	0.001	0.890	0.001	0.010
PU	CE	100.00%	0.014	0.905	0.014	0.024
	CR	96.40%	0.013	0.905	0.013	0.024
PE	CE	100.00%	0.010	0.905	0.011	0.044
	CR	91.10%	0.005	0.905	0.006	0.092
SE	CE	100.00%	0.015	0.905	0.016	0.003
	CR	94.60%	0.013	0.905	0.014	0.003
AP	CE	100.00%	0.011	0.890	0.042	0.103
	CR	96.40%	0.010	0.890	0.059	0.121
DA	CE	100.00%	0.017	0.905	0.019	0.004
	CR	96.40%	0.041	0.905	0.045	0.000
CI	CE	100.00%	0.014	0.890	0.016	0.011
	CR	96.40%	0.012	0.890	0.014	0.012

## Discussion

Focusing on AI-empowered neighborhoods, this study tests multi-path mechanisms through which five experiential cues (R, PU, PE, SE, and AP) reach continued use intention (CI) and well-being (WB) via residents’ digital attachment (DA), integrating triple evidence from PLS-SEM (linear paths and mediation), ANN (non-linearity and non-compensatory effects), and NCA (necessity). From an environmental psychology perspective, AI-empowered neighborhoods can be understood as an ambient socio-digital neighborhood setting embedded in residents’ everyday community life, where interfaces, interaction scripts, and governance processes operate as contextual cues that residents continuously interpret—thereby shaping perceived control, predictability, social climate, and the formation of place-related bonds. Overall, the findings support a layered generative logic of instrumental cognition → affective belonging → behavior/well-being, not as a single route but as a configurational outcome jointly constrained by cue synergy and structural non-linearity.

First, the concordant ordering from SEM and ANN indicates that reliability (R), perceived usefulness (PU), and perceived ease of use (PE) form an instrumental appraisal cluster—“credible–useful–easy”—whose explanatory power for DA clearly exceeds that of service efficiency (SE) and aesthetic perception (AP). This pattern aligns with recent IS evidence: improvements in system/service quality and usability reduce uncertainty, build trust, and in turn support continuance and deeper relational investment (e.g., attachment and stickiness) (Jo, 2022). Meanwhile, efficiency and aesthetics primarily amplify this translation via processing fluency/lower cognitive load and embodied pleasure, but do not by themselves determine attachment strength: enhancements in aesthetics and interface quality activate a fluency → liking → positive affect chain that indirectly fosters subsequent attitudes and relational construction (Popescu and Holman, 2024). In sum, these cues operate as a “cognition–trust base” that bridges to “affect–attachment”: once dependability and attainable outcomes are established (R/PU/PE in place), residents are more willing to invest attention and time in relational dimensions, generating and deepening DA—consistent with evidence on the quality → trust → continuance/stickiness pathway (Jo, 2022).

Second, regarding mediation and cascading effects, DA affects WB both directly and via a significant sequential mediation through CI (DA → CI → WB). Contemporary IS studies show that CI relates positively to users’ subjective well-being and serves as a conduit in technology–experience pathways across contexts (e.g., e-government, intelligent assistants, and digital platforms), supporting a testable attachment → continuance → well-being chain (Razem, 2020). ANN results additionally suggest nonlinear gains of DA and CI in predicting WB: after an instrumental threshold is met (R/PU/PE reasonably satisfied), the marginal contribution of relational investment (DA) rises more steeply, whereas isolated process/aesthetic cues exhibit diminishing or weak returns—echoing recent findings of nonlinear, threshold-like, or very small effects between digital use and well-being, and motivating models that capture nonlinearity (e.g., ANN) (Vuorre et al., 2021). Put differently, a “instrumental assurance → relational amplification → well-being accumulation” cascade fits the data: once credibility, usefulness, and ease are secured, residents sustain engagement and, over time, “deposit” higher WB—also reflected in recent continuance → satisfaction/well-being research (Razem, 2020). Notably, despite the consistent SEM–ANN–NCA evidence supporting the DA → CI → WB chain, the explained variance for CI and WB remains modest (*R*^2^ = 0.162 and 0.214). This is consistent with the multifaceted nature of well-being, which is also influenced by unmodeled factors such as health status, socioeconomic resources, offline social ties, and neighborhood stressors (Yang and Zou, 2025). Accordingly, the present framework should be read as a parsimonious, mechanism-focused model that identifies actionable design levers in AI-enabled neighborhood services, rather than a full explanatory model of residents’ well-being.

Environmental relevance of behavior and VR intervention implications. Although the focal outcomes are continued use intention (CI) and well-being (WB), the findings also have clear environmental-psychological relevance. In AI-empowered neighborhoods, sustained engagement with neighborhood digital services can enable low-carbon service substitution by reducing unnecessary trips, repeated queueing, and paper-based procedures, while supporting resource-efficient community coordination (e.g., appointment-based flow management and timely information nudges) (Xu et al., 2025). Importantly, our results suggest that such environmentally relevant participation is more likely to stabilize when the instrumental assurance bundle (R/PU/PE) is secured, because predictability and controllability lower friction and make repeated engagement feasible. This boundary condition is also pertinent for VR-based neighborhood interventions (e.g., immersive risk-preparedness drills or environmental education modules): VR features may enhance salience and engagement, but durable participation and downstream behavioral spillovers require a reliable, useful, and easy baseline (Qawqzeh et al., 2025; Jung, 2022). Finally, the NCA results imply that environmentally beneficial engagement can emerge via multiple cue configurations, consistent with an equifinality view of behavior change in complex neighborhood settings.

Third, the near-zero NCA effect sizes indicate no single necessary condition, corroborating a sufficiency-and-configuration logic of outcome generation: high WB/CI does not hinge on any solitary threshold but on complementary coupling among multiple cues. Combining ANN importance with SEM’s significant paths, an effective typical configuration is: R/PU/PE reach robust levels to form a trust base; SE and AP provide process–situational boosts; together they raise DA, which—via CI’s temporal extension—converts experiential utility into stable WB. This “sufficient but not necessary” structure helps explain how diverse communities can attain comparable well-being through different combinations.

At the method-integration level, the three evidentiary streams are complementary and mutually checking: SEM identifies directed causal structure and mediation weights; ANN reveals non-linear responses and non-compensatory relations (deficits in some cues cannot be fully “made up” by others); NCA bounds necessity, avoiding the misreading of high importance as a hard threshold. This linear–nonlinear–necessity triangulation strengthens the robustness and interpretability of the findings and extends explanatory insight into how AI-empowered neighborhood experiences translate into person–environment bonding and well-being, offering a reusable multi-method integration template for future research.

### Theoretical implications

#### First, operationalizing socio-digital environmental cues: from a “global tech experience” to five actionable environmental affordances

Rather than treating “smart-community experience” as a single evaluative perception, we conceptualize AI-empowered neighborhood services as part of a socio-digital neighborhood environment and specify five engineerable cues—reliability (R), perceived usefulness (PU), perceived ease of use (PE), service efficiency (SE), and aesthetic perception (AP)—as environmental cues/affordances that structure residents’ everyday interactions. This shifts the analytic focus from “technology adoption” to how digitally mediated neighborhood settings shape lived experience, enabling comparable diagnosis of environmental supportiveness, friction, and aesthetic quality for scenario optimization.

#### Second, refining CAC as a person–environment process: instrumental appraisal versus relational bonding as dual psychological pathways

Within the Cognition–Affect–Conation (CAC) logic, our findings delineate two complementary pathways that map onto environmental psychology’s person–environment perspective: an instrumental appraisal channel (PU/PE/R forming perceived predictability, controllability, and functional support) and a relational bonding channel (digital attachment as affective belonging that stabilizes engagement and well-being). The consistent SEM–ANN ordering indicates that instrumental assurance tends to precede affective investment, and that digital attachment functions as the key hinge through which socio-digital environmental appraisals translate into sustained engagement and well-being, with nonlinear gains suggesting threshold-like amplification once basic environmental assurances are met.

#### Third, interactional reorientation of social presence as a socio-digital “social climate” mechanism

We reframe Social Presence from a media attribute to a situated, co-constructed social climate within neighborhood tasks. In AI-enabled community services, interface cues and response patterns contribute to residents’ perceived being-with-others, being understood, and being supported—core experiential qualities of everyday environments. The five cues provide contextual supports for this climate: enhanced dependability, process fluency, and situational aesthetics reduce processing burden and uncertainty while strengthening felt understanding, thereby facilitating the emergence and accumulation of digital attachment as a stable person–environment bond.

#### Fourth, extending place attachment into socio-digital neighborhoods: digital attachment as a person–environment bond anchoring contextual well-being

We position digital attachment (DA) as an emergent form of person–environment bond within hybrid neighborhood settings and confirm both its direct effect on well-being (WB) and its sequential mediation (DA → CI → WB). This advances community research beyond satisfaction toward a time-based mechanism of belonging → sustained engagement → well-being accumulation, suggesting that repeated engagement with supportive socio-digital environments can sediment relational capital that translates into contextual well-being.

#### Fifth, moving from single-cause accounts to equifinality: complementary cue configurations rather than necessary thresholds

The NCA indicates that no single antecedent constitutes a necessary condition; high WB/CI arises through complementary combinations of environmental cues rather than isolated thresholds. Together with SEM paths and ANN importance ranks, this supports an equifinality logic common in environmental psychology: comparable well-being can emerge from different cue bundles. A diagnostic high-output configuration is that R/PU/PE establish a trust-and-control base, SE and AP provide process–situational gains, and their synergy elevates DA, which—via CI over time—converts environmental utility into more stable well-being.

#### Sixth, methodological contribution for environmental psychology in socio-digital settings: SEM–ANN–NCA triangulation

We offer a transferable three-stage framework suited to socio-digital environmental psychology: PLS-SEM tests measurement quality and linear mechanisms, ANN captures nonlinear and non-compensatory predictive patterns, and NCA clarifies necessity constraints. This triangulation strengthens interpretability by distinguishing “important predictors” from “indispensable conditions,” and provides a reusable template for analyzing how socio-digital environmental cues shape person–environment bonds and well-being outcomes.

### Managerial implications

#### First, prioritize instrumental thresholds before affective conversion

Set reliability (R), perceived usefulness (PU), and perceived ease of use (PE) as baseline “pass lines”—for example, verifiable thresholds for error rates, one-and-done processing, and latency on critical tasks. Evidence indicates that this “credible–useful–easy” foundation is a precondition for cultivating and stabilizing digital attachment (DA). Once these thresholds are met, invest in situational and relational design to lift continued use intention (CI) and well-being (WB).

#### Second, use efficiency and aesthetics as amplifiers, not substitutes

Leverage service efficiency (SE) to reduce process friction (clear feedback, pruning redundant steps, peak-time orchestration), and aesthetic perception (AP) to lower cognitive load and enhance contextual fit (localized visual grammar, clear information hierarchy). Their role is to amplify the conversion from R/PU/PE to DA, and—through accumulated DA—raise CI and WB downstream, rather than directly determining WB in isolation.

#### Third, optimize configurations rather than chasing single-point extremes

Across communities and segments, track a comparable KPI dashboard for R/PU/PE, SE, AP → DA → CI → WB. Use stratified A/B tests and time-sliced analyses to estimate the marginal returns of different cue mixes, avoiding treatment of any single cue as a “necessary condition.” In practice, a common high-yield mix is: robust R/PU/PE, targeted SE bottleneck fixes, and light AP tuning aligned with scenario narratives; DA then drives time-based accumulation in CI, ultimately improving WB.

#### Fourth, embed “minimal participation loops” at key touchpoints

Around high-presence moments (e.g., task completion, issue resolution, and community events), implement lightweight flows of event closure → relational trigger → low-barrier participation (e.g., follow-up care, secondary responses, simple sign-up/subscribe options). This ensures emotions and meaning are promptly captured, allowing DA to sediment over time via CI into more stable WB. Offer deep/shallow entry points for high/low involvement users, reducing churn and improving cross-cycle retention.

#### Fifth, translate engagement into sustainability-oriented participation and VR-enabled programs

Treat CI not only as a usage outcome but as a behavioral gateway for sustainability-oriented community programs (e.g., digital-first procedures, appointment-based traffic reduction, paperless workflows). When deploying VR modules (e.g., environmental communication or preparedness training) in AI-empowered neighborhoods, prioritize R/PU/PE as “participation prerequisites,” then use SE/AP to reduce friction and enhance experiential fit, so that digital attachment can sustain repeated participation and support longer-term behavioral spillovers.

### Limitations and future research

This study examined AI-empowered neighborhood centers and validated the chain instrumental cues (R/PU/PE/SE/AP) → digital attachment (DA) → continued use intention (CI) → well-being (WB) using a SEM–ANN–NCA triangulation. Several limitations remain. First, the sample was drawn from a small set of pilot communities in Eastern China and recruited via volunteers, introducing contextual and self-selection biases that constrain external validity. Second, although a three-wave design helped mitigate common-method bias, the key outcomes (CI, WB) are self-reported; cross-validation with objective behaviors (e.g., actual usage logs, service-ticket closure rates) and third-party assessments was not available.

Future work can proceed along four directions. (1) Cross-region/type comparisons: Employ probability or stratified quota sampling across diverse communities to enhance representativeness, and test structural stability and contextual differences via multi-group SEM and hierarchical models. (2) Multi-source data fusion: Integrate objective and process data (app/miniprogram logs, time-of-day congestion and wait times, service-flow records, event participation logs, sensor-based visitation traces) with self-reports to assess multi-trait matching and convergent/discriminant validity. (3) Longitudinal and causal identification: Use longer panels and causal designs (growth curves/cross-lagged models, staged field A/B tests, regression discontinuity/difference-in-differences) to identify the temporal sequence instrumental thresholds → DA accumulation → time-dependent effects on CI and WB, and to evaluate cumulative impacts of policy or feature iterations. (4) Mechanism expansion and heterogeneity: Incorporate moderators/mediators such as social presence, neighborhood mutual-aid networks, privacy–trust trade-offs, algorithmic fairness, and accessibility for vulnerable groups; combine explainable ML with fsQCA’s configurational lens to map optimal paths and fairness boundaries across population segments × cue configurations. These steps can strengthen external validity while enhancing behavioral verifiability and policy operability.

## Conclusion

This study advances an integrated account of how AI-empowered neighborhoods enhance residents’ community well-being. Drawing on a three-wave panel and a SEM–ANN–NCA triangulation, we show that five designable cues—reliability, perceived usefulness, perceived ease of use, service efficiency, and aesthetic perception—shape well-being predominantly through digital attachment and continued use intention. Instrumental appraisals (credible–useful–easy) form a cognition–trust base that precedes relational investment; digital attachment is the pivotal bridge, exerting a direct effect on well-being and a sequential effect via continuance. ANN results reveal nonlinear, non-compensatory patterns: once instrumental thresholds are met, the marginal returns of relational attachment rise, whereas isolated gains in process efficiency or aesthetics yield limited direct benefits. NCA indicates no single necessary condition, underscoring a configurational logic in which complementary cue bundles—not any solitary factor—deliver high well-being. Beyond technology adoption, the continuance pathway also has potential environmental relevance in AI-empowered neighborhoods, as sustained digital engagement can support low-carbon service substitution and resource-efficient coordination in everyday community governance. In this sense, VR-oriented neighborhood programs (e.g., immersive environmental communication or preparedness drills) may benefit from our findings by treating reliability, usefulness, and ease of use as participation prerequisites and using efficiency and aesthetics as engagement amplifiers.

Managerially, prioritize baseline thresholds for reliability, usefulness, and ease before investing in situational design; treat efficiency and aesthetics as amplifiers that accelerate the conversion from cognition to attachment; monitor cue configurations with comparable dashboards; and embed lightweight participation loops at high-presence touchpoints to consolidate attachment over time. While our multi-site evidence from Eastern China supports generalizable mechanisms, representativeness and self-report outcomes remain constraints. Future work should fuse objective usage/process traces, adopt longer causal designs, and probe heterogeneity (e.g., social presence, privacy–trust, algorithmic fairness). Overall, the findings reposition AI-empowered neighborhood design from single-point optimization to cue synergy, clarifying how instrumental assurance translates into relational amplification and, ultimately, durable well-being.

## Data Availability

The original contributions presented in the study are included in the article/supplementary material, further inquiries can be directed to the corresponding authors.
